# From Single-Ion to Integrated Multi-Ion Platforms: Wearable Sweat Sensors for Electrolyte Monitoring

**DOI:** 10.3390/bios16060317

**Published:** 2026-06-01

**Authors:** Jieru Yang, Junyao Li, Xiao Han, Zewen Wei, Gang Wang, Ting Zou

**Affiliations:** 1 College of Biological Sciences, China Agricultural University, Beijing 100193, China; 2 School of Medical Technology, Beijing Institute of Technology, Beijing 100081, China; 3 Quartermaster Engineering Technology Research Department, System Engineering Institute, Academy of Military Sciences of People’s Liberation Army, Beijing 100010, China

**Keywords:** wearable sensors, sweat analysis, multi-ion monitoring, electrochemical biosensors, optical biosensors, personalized healthcare, flexible electronics, microfluidics

## Abstract

Sweat contains abundant ions, offering a rich source of physiological information for non-invasive health monitoring. Wearable sweat sensors have become a promising technology due to advances in electrochemical devices, sensing materials and structural design. The current monitoring platforms primarily employ two fundamental sensing modalities to convert sweat chemical information into detectable numerical signals: electrochemical (potentiometric, voltammetric, transistor-based) and optical (colorimetric) transduction mechanisms. The demand for more comprehensive physiological and biochemical data in clinical diagnosis and daily health monitoring is driving sensors towards multi-ion detection. Building on these modalities, researchers have optimized hardware and software algorithms based on the characteristics of different ions, thereby promoting the transition of wearable devices from the laboratory to practical applications. Here, we summarize recent progress in wearable sweat ion sensors, focusing on their mechanisms, advantages, and limitations. Finally, current challenges and future prospects of wearable sweat ion sensors for research applications, clinical use, and market demands are discussed.

## 1. Introduction

Sweat is a physiologically rich biofluid comprising a complex milieu of electrolytes, metabolites, hormones, and small-molecule proteins [1]. Within this milieu, ions such as Na^+^, K^+^, Ca^2+^, NH_4_^+^, Cl^−^ and H^+^ play critical roles in modulating biological activities [2,3]. Their concentration fluctuations provide molecular-level insights into hydration status, basal metabolism, and systemic organ function [4]. This provides a unique perspective for the realization of non-invasive, continuous physiological monitoring [5,6]. For instance (Table 1), the quantification of Na^+^, K^+^ and pH is essential for maintaining osmotic pressure and volume across cellular membranes. Thus, real-time assessment of their dynamics is pivotal for evaluating hydration, pre-empting muscle fatigue, and guiding precision rehydration in sports science [7,8,9,10,11]. NH_4_^+^ is often used as a biomarker for the monitoring of liver and kidney functions [12]. Ca^2+^ is intimately involved in neuromuscular function, and its aberrant levels in sweat emerge as potential biomarkers for long-term health tracking [13]. Notably, sweat Cl^−^ concentration has been clinically established as the gold standard for the diagnostic screening of cystic fibrosis [14,15]. Therefore, monitoring of ions in sweat is necessary to offer important data for health management.

Traditional biomarker monitoring relies predominantly on benchtop instruments for ex vivo analysis. While precise, these methodologies are hindered by invasive sampling and operational complexity. More importantly, there is a significant time lag from sampling analysis to result output, making it difficult to capture dynamic changes with seconds in ion concentration in motion or pathological states. Compared with other biofluids, sweat offers advantages such as easier accessibility, lower matrix complexity, and a wider range of choices of sensing materials with the desired biocompatibility, serving as an ideal medium for non-invasive longitudinal monitoring. The emergence of wearable sensor technologies provides a robust solution to these bottlenecks by integrating miniaturized, ref. [16] flexible sensing elements with signal processing and wireless transmission modules, enabling the potential for decentralized monitoring and diagnosis (e.g., at home or point-of-care) attached to the skin surface [1,17].

In recent years, researchers have significantly improved the sensitivity and selectivity of wearable sweat sensors through strategies such as material modification and structural improvement, laying an important foundation for the development of this field. However, different ions in sweat vary in both their concentration ranges and the physiological information they convey, which determines that the detection process needs to accommodate diverse dynamic ranges and application scenarios. Therefore, systematically reviewing sensor design and performance optimization strategies from the perspective of ion characteristics may achieve multiple-parameter, high-precision detection.

**Figure 1:**
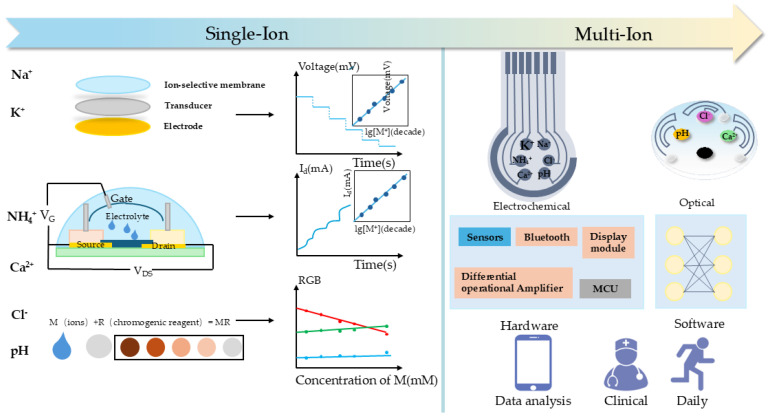
Integration strategy of wearable sweat ion sensor. Single-ion detection mechanism and multi-ion integration strategy. The platform simultaneously analyzes various sweat analytes, including Na^+^, K^+^, Ca^2+^, NH_4_^+^, Cl^−^, and pH. It can be used for clinical and daily life monitoring.

With the increasing demand for personalized precision medicine and comprehensive daily monitoring, sweat sensing technology is advancing toward simultaneous multi-ion detection and multiple-parameter integrated analysis, aiming to achieve an accurate interpretation of complex physiological states (Figure 1). Based on this consideration, this review systematically summarizes the design strategies and recent research progress of various ion sensors, with the goal of providing more targeted technical analysis. By rationally selecting target analytes according to specific application scenarios and employing electrochemical or optical methods for multi-ion signal acquisition, device performance is enhanced through hardware design and algorithm optimization. This enables dynamic tracking of multiple biomarkers and enhances the correlation analysis among physiological signals. This provides a new technological pathway for clinical diagnosis and daily health management.

**Table 1:** Physiological relevance and application potential of sweat ions.

Ion	Range (mM)	Physiological Correlation	Potential Applications	Ref.
Na^+^	10–100	Speculative: Hyponatremia, hypernatremia, dehydration	Sports science, health monitoring	[7,9,10]
K^+^	1–24	Speculative: Muscle activity, hypo- or hyperkalemia	Sports science, health monitoring	[5,18,19,20,21]
NH_4_^+^	0.5–8	Speculative: Exercise intensity, liver and kidney functions	Sports science, health monitoring	[22,23]
Ca^2+^	0.5–3	Speculative: Muscle contraction, bone metabolism	Sports science, health monitoring	[24,25,26,27]
Cl^−^	10–100	Well-established: Dehydration, fatigue	Clinical diagnosis	[28,29]
pH	4–7	Speculative: Acid-base equilibria	Sports science, health monitoring, Clinical diagnosis	[30,31]

Note: Speculative: based on limited evidence, or unresolved mechanistic understanding; Well-established: supported by independent studies with clinical validation.

## 2. Fundamental Sensing Modalities

**Figure 2:**
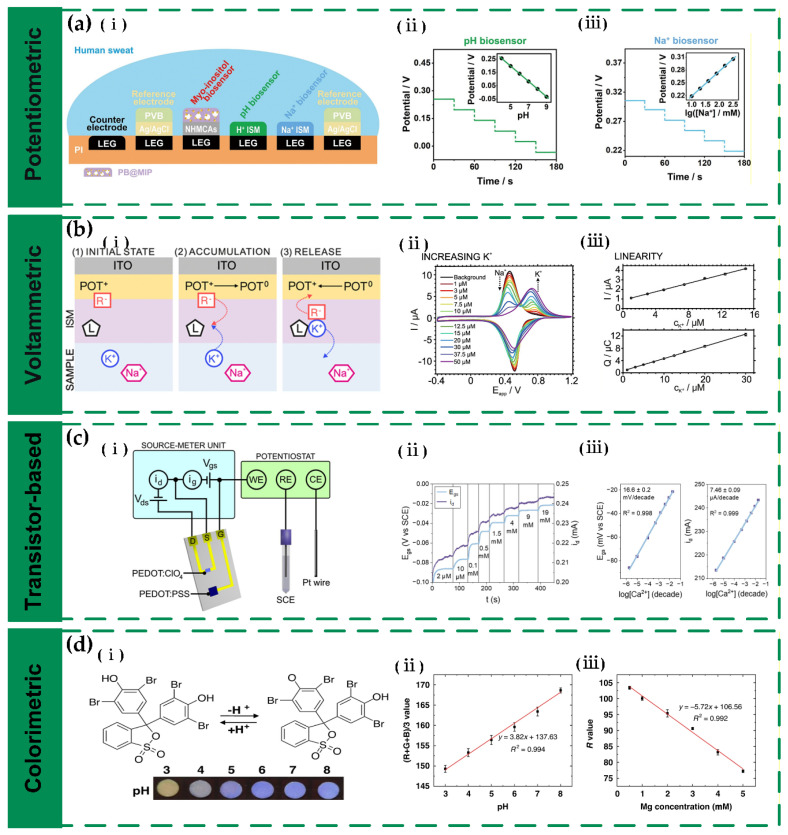
Working mechanisms, performance metrics, and analytical outputs of wearable sweat ion sensors. (**a**) Potentiometric sensing modality: Schematic illustration of the potentiometric transduction architecture (i), with representative open-circuit potential (OCP) responses and the concomitant calibration plots for Na^+^ (ii) and Na^+^ (iii) sensors [32]. Copyright 2025. Reproduced with permission from the American Chemical Society. (**b**) Voltammetric sensing modality: Schematic representation of the voltammetric recognition mechanism (i), alongside the corresponding CV results for the third scan. (ii) Linearity of the peak current and charge with the K^+^ concentration in the sample (iii) [33]. Copyright 2024. Reproduced with permission from the American Chemical Society. (**c**) Organic electrochemical transistor (OECT) platform: Schematic of the transistor-based sensing configuration (i), featuring the real-time transduced signal for Ca^2+^ (ii). Calibration plots obtained using potential and current-based data (iii) [34]. Copyright 2025. Reproduced with permission from the American Chemical Society. (**d**) Colorimetric sensing modality: Schematic of the chromogenic reaction mechanism (i), showing the visual detection results for pH (ii) and Mg^2+^ (iii) [35]. Copyright 2023. Reproduced with permission from Springer Nature.

Novel sweat sensing platforms leveraging diverse analytical methodologies have garnered significant attention for the non-invasive, in situ, and real-time quantification of biomarkers in personalized healthcare and athletic monitoring. The practical deployment of these sensors necessitates a high degree of miniaturization so that they can be worn on the body, while maintaining reliable stability in the complex environment of the complex skin interface. Furthermore, superior analytical metrics, which are characterized by low limits of detection (LOD) and expansive linear dynamic ranges, are required to accurately resolve ionic species across their broad physiologically relevant spectra. Currently, the state of the art in wearable ion sensing is predominantly predicated on electrochemical and optical transduction mechanisms (Figure 2).

### 2.1. Electrochemical Potentiometric Sensing

Potentiometric sensors are extensively utilized for the quantification of ionic concentrations, owing to their exceptional selectivity and rapid response kinetics. These platforms typically comprise a reference electrode (RE) that maintains a stable potential across various solutions and a working electrode (WE) whose potential fluctuates concomitantly with the target ion activity. The determination of ionic levels is thus achieved by measuring the open-circuit potential (OCP) between this electrode pair. As illustrated in Figure 2a, a representative WE architecture features a tri-layer sandwich configuration, from the substrate up to an electronically conductive base layer, an ion-to-electron transduction layer, and a functional ion-selective membrane (ISM). Specific recognition is facilitated by ionophores embedded within the ISM, such as sodium ionophore X for Na^+^ or ETH 129 for Ca^2+^ sensing, which selectively bind target ions to induce a potential shift, effectively transducing the ionic concentration into a measurable open-circuit voltage signal. The mathematical correlation between the transduced potential and the ionic activity is rigorously defined by the Nernst equation:

(1) $$E=E_{0}+\;\frac{RT}{zF}\cdot \ln a$$

where *E* is the measured potential, *E*_0_ is the standard potential at *a* = 1, *R* is the universal gas constant, *T* is the absolute temperature, *z* is the charge number of the ion, *F* is the Faraday constant, and *ɑ* is the activity of the target sweat ion. According to the Nernst equation, the potential signal has a log-linear relationship with the target ion concentration. Accordingly, under the 25 °C condition, the theoretical Nernst limit is 59.16 mV/dec for monovalent ions and 29.58 mV/dec for divalent ions. However, the sensitivity observed in experiments deviates from the theoretical value by ±0.1–0.5 mV·dec^−1^ due to membrane inhomogeneity, such as uneven ionic distribution and interfacial potential fluctuations. The basic transduction mechanism of the potential sensor essentially limits the variation in its sensitivity, although slight disturbances may occur due to temperature-dependent thermodynamic fluctuations.

### 2.2. Electrochemical Voltammetric Sensing

Voltammetric sensors typically operate via a three-electrode configuration, integrating a working electrode (WE) for target recognition, a counter electrode (CE) serving as a current source, and a reference electrode (RE) to maintain a stable electrochemical potential [4,36]. Under an applied bias, electrochemical reactions at the WE interface elicit current signals, where the resulting current-potential profiles are utilized for the precise quantification of target analytes [37,38]. When quantifying electron transfer during interfacial redox processes, voltammetry is particularly amenable to the detection of electroactive species or indirect sensing based on redox-active transitions. However, Na^+^, K^+^, Ca^2+^, and Cl^−^ ions do not possess the properties of redox reactions that are easily occurring and reversible in aqueous solutions, making them difficult to detect directly by conventional voltammetry. Currently, voltammetry is commonly used to detect ammonium and hydrogen ions [12,39,40,41].

### 2.3. Transistor-Based Sensing

Emerging platforms, such as organic electrochemical transistors (OECTs) and ion-sensitive field-effect transistors (ISFETs), incorporate semiconductor physics, which enables analyte quantification at low operating voltages. Architected with source, drain, and gate terminals, these devices function by the target ions in sweat recognition or penetrate the channel, and they cause small changes in the potential at the gate–electrolyte interface, resulting in a channel current response for target quantification [42,43]. The inherent compatibility of these platforms with microfabrication facilitates seamless miniaturization and the deployment of high-density multiplexed sensing arrays [44,45].

While some studies claim super-Nernstian responses in transistor-based sensors [46], these phenomena typically arise from coupled redox reactions or non-equilibrium kinetic conditions, rather than an intrinsic amplification of the interfacial potential. The transconductance effect merely enhances signal transduction in current readout and does not physically change thermodynamic limits. Therefore, such improvements in apparent sensitivity should not be conflated with the intrinsic response characteristics of the sensing interface.

### 2.4. Optical Colorimetric Approaches

Beyond electrochemical means, wearable optical sensors have been extensively investigated for the longitudinal monitoring of electrolytic species [47]. These optical platforms are frequently integrated with microfluidic architectures to facilitate continuous sweat sampling and efficient fluidic exchange [48,49]. Colorimetric sweat sensors primarily operate via chemochromic transduction mechanisms. These mechanisms are generally categorized as follows: the formation of chromogenic coordination complexes through ion chelation with specific ligands (e.g., for chloride), and color changes induced by pH dependent acidic or alkaline shifts [31,50]. The analytical output can be quantified through visual comparison with a standard color card, or through automatic chromatic data extraction and ion analysis using digital imaging analysis on a smartphone [4]. While utilizing flexible, lightweight substrates such as paper or polymers alleviates fabrication costs and wearer burden, the analytical precision remains vulnerable to fluctuations in ambient illumination, skin pigmentation, and algorithmic variations, thereby precluding high-frequency, continuous real-time monitoring.

## 3. Advanced Sensing Platforms for Sweat Ion Monitoring

Inorganic ions in sweat, including Na^+^, K^+^, Ca^2+^, NH_4_^+^, Cl^−^ and H^+^, are integral to fundamental physiological processes, with their concentration dynamics providing key predictive insights into various health metrics, such as hydration assessment and early warning of electrolyte imbalances in diverse clinical contexts. Consequently, the development of wearable ion selective sensors for the in situ monitoring of these inorganic species has emerged as a prominent research frontier in the field of wearable electronics. Current research endeavors are primarily concentrated on the optimization of electrochemical potentiometric and optical colorimetric platforms, the synthesis of advanced responsive materials, and the synergistic integration of microfluidic sampling modules with flexible circuitry, alongside advancements in wireless transmission architectures and data-driven algorithmic analysis.

### 3.1. Monitoring Sodium Ion in Sweat (Na^+^)

As a predominant electrolyte in perspiration, Na^+^ serves as a critical biomarker for evaluating body fluid balance, electrolytic homeostasis, and heat stress [6], and monitoring Na^+^ levels is essential for assessing dehydration risks and preventing hyponatremia caused by sweat [9,10]. In addition, pH plays a key role in the homeostasis of human body fluids [11], and the synergistic monitoring of these multi-parameter indicators has important potential for applications in sports medicine and personalized healthcare (Table 2).

Solid-state Na^+^-selective electrodes based on potentiometry have been extensively deployed in sweat analysis due to their exceptional selectivity. Conventional sensors typically utilize the conducting polymer PEDOT:PSS as the transduction layer. However, this architecture is prone to the formation of an unstable water layer at the interface between the ion-selective membrane (ISM) and the solid contact, leading to significant potential drift and compromised accuracy. To achieve long-term stability without performance degradation, researchers [51] engineered a novel SnS_2_-MoS_2_ heterojunction as the transduction layer. By constructing a mesoporous heterostructure with a large surface area of 186.31 m^2^/g, the authors leveraged the 699 μF interfacial capacitance to suppress potential drift to an exceptional 1.37 μV/h. Furthermore, the 132° contact angle effectively precludes water infiltration, eliminating the formation of the interfacial electrolyte layer. This transduction layer yielded a Nernstian sensitivity of 57.86 mV/dec and a low detection limit of 10^−5.7^ M, demonstrating robust resistance to O_2_/CO_2_ interference and providing a reliable solution for long-term monitoring in sports medicine and chronic disease management. Additionally, the formation of electrical double layers at the interface can induce screening effects, leading to sluggish response times. To address this, Chen et al. [52] proposed the application of continuous voltage pulses to the control gate (CG) of floating-gate OECTs (FG-OECT) to modulate sensing performance. Specifically, applying pulsed potentials to the second floating gate (FG2) electrode promotes ion drift and migration into the ion traps within the ISM and solid electrolyte layers. The captured ions modulate the electronic flow, thereby disrupting the initial charge balance on the FG2 surface and establishing a new equilibrium. With a response time reduced to below 0.1 s, the device maintains high transconductance performance. The device exhibits an exceptional sensitivity of 345 μA/dec for Na^+^ across a physiologically relevant concentration range from 10^−5^~10^−1^ M. To achieve long-term, stable Na^+^ monitoring, it is imperative to not only enhance the intrinsic stability of the sensor but also address the critical challenge of sustainable power delivery. Researchers integrated a flexible, quasi-two-dimensional perovskite solar cell (quasi-2D FPSC) module as a robust self-powered device [53]. Under low-intensity indoor illumination (200 lux), this FPSC module delivers continuous energy to the Na^+^ sensing system (power consumption: 0.8 mW/cm^2^); with a minimal efficiency decay of 7.3% after 30 days of operation, this strategy effectively circumvents the maintenance bottlenecks associated with frequent coin-cell battery replacements. Ultimately, a 12 h real-time monitoring trial conducted under mixed indoor and outdoor lighting conditions further validated the system’s exceptional endurance and environmental robustness.

**Table 2:** Monitoring of Na^+^, K^+^, and pH in sweat.

Ions	Methods	Substrate	Electrode Fabrication	Electrode Material	Transducer	ISM Polymer	Reference Membrane	Detection Range (mM)	Sensitivity (μA/dec, mV/dec)	Response Time	Ref.
Na^+^	ISFET (Potentiometry)	Silicon on Insulator	CMOS Photolithography	Si_3_N_4_ Gate	Field Effect (Si_3_N_4_)	Ebecryl photocurable polymer + PVC	PVB	0.032–1000	60.7 mV/dec	N/A	[6]
Na^+^	Potentiometry	Glassy Carbon	Drop-casting	Glassy Carbon	SnS_2_−MoS_2_	PVC	N/A	0.001–100	57.86 mV/dec	<10 s	[51]
Na^+^	Potentiometry	Thermoplastic Polyurethane	Screen Printing	TPU/Exfoliated Graphene Flakes	TPU/EGF	PVC	PVB/NaCl	0.1–100	58.3 mV/dec	9.6 s	[54]
Na^+^	Potentiometry	Cotton Thread/Textile	Dip-coating and Electrodeposition	Carbon Black-coated Cotton	Sodiated Carbon-PEG/PEDOT:PSS/Ti_3_C_2_T_x_ Mxene	PVC	PVC/ETH500	0.12–120	58.4 mV/dec	N/A	[55]
Na^+^, pH	Potentiometry	Polyester (Electrodes)/Paper (Fluidics)	Screen Printing	Graphite Ink	Carbon Black (Na^+^); Iridium Oxide (pH)	PVC	PVB	Na^+^: 1–1000; pH: 4–7	Na^+^: 56 mV/dec; pH: −80 mV/pH	<30 s	[56]
Na^+^, K^+^	OECT	Polyimide	Magnetron Sputtering and Spin-coating	Gold	PSS:Na (Polyelectrolyte on FG)	PVC	N/A	0.01–100	Na^+^: 412 μA/dec; K^+^: 345 μA/dec	<0.1 s	[52]
Na^+^, pH	Potentiometry	PET	Screen Printing	Carbon Ink	PEDOT:PSS (Na^+^); Polyaniline (pH)	PVC	PVB/NaCl	Na^+^: 5–160; pH: 4–8	Na^+^: 57.71 mV/dec; pH: 59.99 mV/pH	Real-time	[57]
K^+^	Potentiometry	Fabric (Polyester warp, PPVN weft)	Electro-assisted core spinning (EACST)	Nylon yarn (Core)	PAN/PVP/Valinomycin Nanofibers	Integrated into nanofibers	Ag/AgCl	0.01–100	34.7 mV/dec	2.1 s	[58]
K^+^	Potentiometry	Screen-Printed Electrode (SPE)	In situ polymerization	Carbon	PPy Nanofibers (CuPcTs doped)	PVC/DOS	Ag/AgCl	0.05–100	62 mV/dec	17 s	[59]
K^+^	Potentiometry	Cotton yarn	Wet-spinning	PANI/SWCNTs	PEDOT:PSS	PVC/DOS	Pseudo-reference	2–32	41.9 ± 0.7 mV/dec	Real-time	[60]
K^+^	Potentiometry	PET	Screen Printing/Stencil Printing	Carbon/Carbon Black	Carbon Black	PVC/DOS	P(BMA-co-MM)/KCl	10^−4^–10^−1^ M	56.1 ± 0.7 mV/dec	<11 s	[61]
K^+^	Potentiometry	PET	Screen Printing	rGO-modified Carbon Ink	N,S-doped Holey Graphene/MXene/TiO_2_	PVC/DOS (K: Valinomycin)	PVB/NaCl	0.19–125	58.7 mV/dec	Real-time	[62]
K^+^	OECT	PET (Sensor) + Textile (Fluidics)	Screen Printing	Carbon (S/D), Ag/AgCl (Gate)	PEDOT:PSS	PVC/DOA	Ag/AgCl (Gate)	0.1–100	−119 µA/dec	Real-time	[63]
K^+^, pH	Potentiometry	PET	Screen Printing	Conductive Carbon Ink	Zn-MOF-derived Porous Carbon Nanorods (MPCNs)	PVC/DOS (K: Valinomycin)	Ag/AgCl/PVB	K^+^: 1–100	K^+^: 55.0 mV/dec pH: 65.8 mV/pH	Real-time	[64]
K^+^, pH	Potentiometry	Carbon Fiber Thread (Yarn)	Coaxial Wet-spinning/Acid-etching	Carbon Fiber/PEDOT:PSS	PEDOT:PSS (Core)	Porous PU/Valinomycin	Ag/AgCl/PVB	1–32	K^+^: 54.89 mV/dec pH: 40.2 mV/pH	10–13 s	[65]
K^+^, pH	Potentiometry	PET	Printing	Conductive Carbon ink	β-CD/RGO (β-cyclodextrin functionalized graphene)	PVC/DOS (K: Valinomycin)	AgCl/CC ink/REM	K^+^: 10^−4^–10^−1^ M	K^+^: 55 mV/dec pH: 51 mV/pH	Real-time	[66]
Na^+^, K^+^	Potentiometry	Polyimide	Screen Printing	Au	Au (Direct)	PVC/DOS	PVB/Ag/AgCl	Na^+^: 5–160 K^+^: 1–32	Na^+^: 43.76 mV/dec K^+^: 57.38 mV/dec	Real-time	[67]
Na^+^, K^+^	Potentiometry	Polyimide	Photolithography/Sputtering	Au	PEDOT:PSS	PVC	PVB/MWCNTs	Na^+^: 10–160 K^+^: 1–32	Na^+^: 87.9 mV/dec K^+^: 48 mV/dec	~60 s	[68]
Na^+^, K^+^	Liquid-Gated Transistor (LGT)	PET	Laser Ablation	Au	Reduced Graphene Oxide (rGO)	PVC/DOS	Gate Electrode (Au)	0.01–100	Na^+^: 1 µA/dec K^+^: 1 µA/dec	5–15 s	[69]
Na^+^, K^+^	Potentiometry	Nonwoven polyester fabric	Screen Printing	Graphene	PLL@Fe_3_O_4_/Gr Heterostructure	Bio-inspired Sea-Island structure	Ag/AgCl	1–160	Selectivity Coeff α ≈ 1.45	2.8 s	[70]
Na^+^, K^+^	Potentiometry	PET (Electrodes) + Paper (Fluidics)	Screen Printing + Wax Printing	Carbon paste	Electrodeposited AuNPs	PVC/DOS	PVB/Ag/AgCl	Na^+^: 10–160 K^+^: 2–32	Na^+^: 54.29 mV/dec K^+^: 102.5 mV/dec	Real-time	[71]
Na^+^, K^+^	Potentiometry	PDMS/PVDF	Vacuum Filtration Transfer Printing (PS-VFTP)	SWCNTs	SWCNTs-Pt-PEDOT	PVC/DOS	Ag/AgCl	Na^+^: 10–160 K^+^: 1–32	Na^+^: 60 mV/dec K^+^: 58 mV/dec	<1 s	[72]
Na^+^, K^+^	Potentiometry	Printed Circuit Board (PCB/FR4)	Electroless Nickel Immersion Gold (ENIG)	Gold	PEDOT:PSS	PVC/DOS	PVB/NaCl	Na^+^: 10^−5^–10^−1^ M K^+^: 10^−4^–10^−1^ M	Na^+^: 56.5 mV/dec K^+^: 65.7 mV/dec	23.9 s (Na^+^) 30.9 s (K^+^)	[73]

Note: Sensitivity units vary based on the sensing mechanism. Values in μA/dec represent amperometric sensitivity, while values in mV/dec represent potentiometric sensitivity. These metrics are not directly comparable numerically across different sensing principles. For the purpose of comparison in this review, “Real-time” is quantized as a response time < 30 s in this table. N/A, not applicable.

### 3.2. Monitoring Potassium Ion in Sweat (K^+^)

Potassium ions (K^+^) are indispensable for maintaining cellular metabolism and modulating fundamental neuromuscular functions [5,18]. Deficient K^+^ levels serve as a primary indicator of dehydration, potentially eliciting muscle spasms [19,20], asthenia, and fatigue [21]. Conversely, elevated K^+^ concentrations can induce hyperkalemia, leading to severe cardiac arrhythmia or even sudden cardiac arrest [22]. Recent advancements in the real-time quantification of K^+^ provide critical diagnostic data for the preventive management of disorders arising from ionic imbalances.

The flexibility of wearable sweat sensing devices is very important. Based on this, textiles characterized by their excellent breathability and wearer comfort have become typical substrates for flexible devices. With progressive research, textile-based electrochemical sensors have undergone significant evolution. For these platforms, the mechanical flexibility and bending endurance of the sensing electrodes are of paramount importance. Researchers [58] developed a core-sheath sensing yarn (PPVN) utilizing a novel electro-assisted core spinning technology (EACST), architecting a polyacrylonitrile (PAN)/polyvinylpyrrolidone (PVP)/valinomycin nanofiber sheath over a structural nylon yarn core. This functional yarn was woven with superhydrophobic polyester to fabricate the PPVN-EFS electrochemical textile sensor. By leveraging superior hydrophilicity and the high specific surface area of the sheath nanofibers, this platform achieved a rapid response time of 2.1 s for K^+^ and sustained operational stability for over 6000 s. Addressing the technical bottlenecks in large-scale manufacturing, Ma et al. [70] employed high-throughput screen-printing to deposit functionalized inks onto non-woven polyester, constructing a biomimetic “sea-island” electrostatic heterostructure comprising a graphene network and PLL-modified nanoparticles. This design leverages a spatial electrostatic gradient induced by poly-L-lysine-modified Fe_3_O_4_ nanoparticles on graphene to enhance the Na^+^/K^+^ selectivity coefficient to 1.45 across a broad linear range (1–160 mM), demonstrating the viability of low-cost, industrial-scale fabrication via advanced surface engineering. Additionally, extensive efforts have been dedicated to integrating storage functionality. Galliani et al. [63] utilized stereolithography (SLA) 3D printing to define resin microstructures within the interstitial spaces of textile fibers, effectively transforming disordered pores into controllable microfluidic networks. Through a vertically folded architecture exploiting capillary pressure gradients, the system enables the regulated collection, storage, and transport of sweat within the textile matrix, yielding a linear K^+^ sensitivity of −119 μA/dec. This advancement significantly augments the fluid-handling capabilities of textile sensors, evolving them into sophisticated sampling platforms. To achieve complete energy autonomy, Tong et al. [60] engineered sweat-activated batteries by weaving PANI/SWCNT composite fibers into the textile structure. The yarns function as both separators and salt bridges, facilitating efficient fluid transport through microscale channels via capillary forces. This self-powered system utilizes exercise-induced perspiration to energize the onboard electronics, enabling the real-time detection of K^+^ fluctuations within the 11.7–12.9 μmol L^−1^ range.

### 3.3. Monitoring Calcium Ion in Sweat (Ca^2+^)

Serving as a strong indicator, calcium ions are fundamentally integral to a myriad of vital physiological processes, including intracellular signal transduction, muscular contraction, skeletal metabolism, and neurotransmitter exocytosis. Elevated concentrations of Ca^2+^ in perspiration can elicit deleterious systemic effects, potentially inducing acid-base imbalances, hypercalciuria-associated complications, and cirrhosis [13]. Conversely, its excessive depletion signifies a heightened risk of clinical hypocalcemia [25,74].

Nevertheless, the relatively low physiological spectrum of Ca^2+^ in sweat (0.5–3 mM) necessitates the engineering of sensing platforms with ultra LOD to precisely resolve transient ionic fluctuations. Ion-selective electrodes (ISEs) currently represent the benchmark methodology for the quantification of calcium species (Table 3). Liu et al. [75] developed a composite membrane electrode material utilizing polydopamine-functionalized reduced graphene oxide (PtPRGO). By exploiting the intrinsic reductive and adhesive properties of polydopamine, the researchers uniformly anchored platinum nanoparticles within graphene interlayers, architecting a robust three-dimensional (3D) layered hierarchical structure. This architecture significantly augments the effective surface area and enhances the permeability of the ion-selective solution, while simultaneously precluding membrane delamination and maximizing interfacial contact with the PtPRGO to accelerate both ionic diffusion and electronic charge transfer. Consequently, the Ca^2+^ sensor achieved a super-Nernstian sensitivity of 91.98 mV/dec with sustained operational stability for up to 9.8 h. However, such super-Nernstian may result from ion-electron coupling transport initiated by Pt nanoparticles or RGO, or non-selective ion permeability caused by insufficient membrane stability. These factors may amplify the potential response while weakening ion selectivity Therefore, when evaluating the potential of this type of sensor for clinical application, it is necessary to focus on the in-depth analysis of the response mechanism and the strict verification of selectivity, rather than merely relying on the enhancement of apparent signals as the basis for judging performance. Beyond improving performance through device interface engineering, signal processing methods can also achieve robust responses under chemical interference without increasing device complexity. To this end, researchers [76] introduced a wearable multiparameter electrochemical signal detection system (WMEDS) designed for the sophisticated processing of multi-analyte signals in sweat. Within the Ca^2+^ concentration range of 0.5–4 mM, the WMEDS exhibited high fidelity, with a negligible average deviation of 3.6 mV when benchmarked against standardized commercial electrochemical workstations.

### 3.4. Monitoring Ammonium Ion in Sweat (NH_4_^+^)

The secretion of ammonium (NH_4_^+^) in perspiration is fundamentally coupled with protein catabolism and metabolic energetic yield [17]. Hepatic dysfunction precludes the efficient conversion of ammonia into urea, rendering elevated sweat ammonium levels a critical prognostic indicator for liver-related pathologies [23].

Traditional ISEs predicated on ion-selective membranes (ISMs) containing organic ionophores are frequently plagued by inherent biotoxicity, prohibitive costs, and suboptimal mechanical robustness (Table 4). To circumvent these limitations, Tang et al. [79] proposed an ISM-free potential sensing strategy based on hybrid electron–ion conductor tungsten bronze (K_x_WO_3_/Na_x_WO_3_). W^6+/5+^ is the redox couple for ion-to-electron transduction. K_0.3_WO_3_ detects NH_4_^+^ through lattice ion exchange within the hexagonal tungsten bronze framework (NH_4_^+^ replacing K^+^), achieving a sensitivity of 58.1 mV dec^−1^, with a potential drift of only 0.08 mV h^−1^ over 25 h. NaWO_3_ achieves a H^+^ selective response via the surface H_x_WO_3_ hydrated layer, while the bulk NaWO_3_ acts as an electron conductor. A flexible dual-electrode sensor integrated on PET can simultaneously record NH_4_^+^ and pH responses without mutual interference, effectively avoiding the water-layer drift and biotoxicity issues of traditional ISEs. Although the concept of ISM-free has been proposed, the potential response of the sensor is limited to single-ion solution tests, and no cross-sensitivity verification has been conducted. The lack of a cross-sensitivity matrix and error propagation analysis makes it impossible to determine signal independence or the need for decoupling. Therefore, the sensor array can currently only be regarded as sensing separately, rather than performing simultaneous quantitative measurements. In addition, Patil et al. [12] employed a voltammetric approach using Ag-CuO-MGCN nanocomposites to facilitate the formation of [Cu (NH_3_)_4_]^2+^ complexes, enabling an ultra-sensitive, nanomolar method for NH_4_^+^ detection without a polymer membrane.

Furthermore, while nonactin is a ubiquitous NH_4_^+^ ionophore, its selectivity is often compromised by K +interference due to similarities in ionic radii and valency. To address this crosstalk, the NH_4_^+^/K^+^ dual sensor array was integrated, and a cross-calibration algorithm to suppress K^+^ interference, optimizing the NH_4_^+^ selectivity coefficient to 0.11 [80]. Across a dynamic range of 1–100 mM, the system maintained a sensitivity of 58.7 mV/dec and achieved an 18% enhancement in analytical precision post calibration, demonstrating the versatility of this biocompatible platform for NH_4_^+^ analysis in blood, sweat, and tears.

**Table 4:** Monitoring of NH_4_^+^ in sweat.

Ions	Methods	Substrate	Electrode Fabrication	Electrode Material	Transducer	ISM Polymer	Reference Membrane	Detection Range (mM)	Sensitivity (μA/dec, mV/dec)	Response Time	Reference
NH_4_^+^	Potentiometric	PDMS/PU	Screen printing and Electrodeposition	Carbon ink	3D Graphene Oxide-CNT network	PU/PVC	PVB/NaCl	0.001–100	59.6 mV/dec (0% strain) 42.7 mV/dec (40% strain)	N/A	[81]
NH_4_^+^, pH	Potentiometric (ISM-free)	PET	Magnetron sputtering and Drop-casting	Au	NH_4_^+^: K_x_WO_3_ pH: NaWO_3_	None (ISM-free)	PVC/KCl^−^Ag/AgCl	NH_4_^+^: 0.01–100	NH_4_^+^: 58.1 mV/dec pH: 50.1 mV/pH	<15 s	[79]
NH_4_^+^, K^+^	Potentiometric	PTFE	Thermal evaporation and Electrodeposition	Au	PEDOT: PSS	PVC/DOS	PVB/NaCl	NH_4_^+^: 0.001–100 K^+^: 1–20	NH_4_^+^: 58.7 mV/dec K^+^: 61.5 mV/dec	Real-time	[80]
NH_4_^+^	Potentiometric	PI	E-beam evaporation and Photolithography	Au	PEDOT: PSS	PVC/DOS	PVB/NaCl + MWCNT	NH_4_^+^: 2.5–40	NH_4_^+^: 60.3 mV/dec pH: 55.3 mV/pH	~4 min	[82]
NH_4_^+^	Potentiometric	PET	Screen printing	Carbon ink	CNTs-COOH	PVC/DOS	PVB/NaCl + TPU	NH_4_^+^: 10–100	55.36 mV/dec	200 s	[17]
NH_4_^+^	Voltammetry (DPV)	Glassy Carbon/Paper SPE	Drop-casting/Screen printing	Carbon	Ag-CuO-MGCN	None	Ag/AgCl	0.00001–2 (Low range optimized)	0.184 μA/μM	N/A	[12]
NH_4_^+^**,** Na^+^	OECT	PI	Inkjet printing	Au	PEDOT: PSS	PVC	Ag/AgCl	10^−4^–10^−1^ M	0.91 mA/dec (SI) 130 mV/dec (SV)	Real-time	[83]
NH_4_^+^, Na^+^, K^+^	Potentiometric	PI	Inkjet printing	Carbon	Carbon	PVC-SEBS	PVB/NaCl	NH_4_^+^: 0.5–8 Na^+^: 10–160 K^+^: 2–32	Nernstian	<30 s	[84]

Note: For the purpose of comparison in this review, “Real-time” is quantized as a response time < 30 s in this table. N/A, not applicable.

### 3.5. Monitoring Chloride Ion in Sweat (Cl^−^)

In clinical practice, sweat chloride levels serve as the hallmark diagnostic gold standard for Cystic Fibrosis (CF). CF patients exhibit elevated chloride concentrations (where <40 mM is diagnostic of normal function and >60 mM indicates CF; the 40–60 mM range signifies borderline risk), often accompanied by increased sweat alkalinity [15]. Consequently, the synergistic quantification of chloride and pH emerges as a pivotal tool for CF screening. Among various detection methods, the optical colorimetric approach is particularly suited for resource-limited settings due to its simple setup, ease of operation, and intuitive visual readout (Table 5).

The realization of wearable colorimetric sensors is critically dependent on the selection of responsive substrates. Among various stretchable polymers, hydrogels have emerged as ideal candidates due to their intrinsic flexibility, skin-adhesiveness, and elasticity. Consisting of three-dimensional cross-linked polymeric networks, hydrogels possess exceptional biocompatibility, mechanical robustness, and superior water-retention capabilities, providing irreplaceable merits for sweat sensing applications. He et al. [85] utilized a PVA-Suc hydrogel matrix that effectively precludes reagent leakage. Its high optical transparency ensures unhindered colorimetric readouts, facilitating robust data interpretation and long-term detection of Cl^−^ over a broad range (0–100 mM).

However, since the analyte and the colorimetric reagent in the colorimetric response continue to react over time, the signal has a cumulative nature, and its intensity depends simultaneously on the concentration of the analyte and the duration of the reaction This cumulative characteristic makes it difficult for sensors to distinguish the features of sweat at different time points. Because fresh and unreacted sweat mixed with reacted sweat can lead to inaccurate concentration detection, which seriously hinders the continuous dynamic monitoring of sweat components. To address this bottleneck, a spatial for time compensation strategy has been pioneered using microfluidic architectures that enable real-time tracking of sweat secretion rates and chemical concentrations. Mishra et al. [86] engineered a 200 μm thick collapsible membrane architecture within a pump chamber. This precision-engineered valve facilitates sweat transport exclusively under user-induced negative pressure. This design allows for discrete longitudinal analysis of sweat while maintaining a lightweight, wearable form factor, and such segmented collection techniques provide the foundational framework for precise data analytics.

Regarding data processing, conventional colorimetric quantification relies heavily on RGB (Red, Green, Blue) analysis and traditional statistical methodologies. Driven by the rapid evolution of artificial intelligence, machine learning and deep learning algorithms now demonstrate superior capabilities in capturing multi-dimensional physiological signals and predicting clinical trends, advancing wearable sensing toward intelligent, autonomous, and high-fidelity data interpretation. Researchers [87] introduced a deep learning framework based on YOLOv5 for colorimetric data analysis, utilizing predictive bounding boxes and RGB values for the formalized representation of detection outputs. This methodology enables the accurate classification and quantification of three biomarkers in sweat, yielding LODs of 15.80 μM for Fe^2+^ and 13.90 mM for Cl^−^. These results underscore the technical prowess of AI algorithms in facilitating automated signal recognition and the simultaneous quantification of complex multi-analyte systems.

However, it is important to note that the gold standard for clinical CF screening is still Coulomb titration or electrochemical ISEs. The colorimetric Cl^−^ sensors reported so far are mostly in the research prototype stage and have not yet been translated into clinical routine diagnostic tools. In terms of performance, the response time of the colorimetric method is usually limited by the equilibrium and diffusion process of color development reaction, which takes several minutes. In contrast, electrochemical sensors require a response of seconds. Considering the high requirements of sweat detection induced by pilocarpine iontophoresis to capture dynamic component fluctuations, and improve detection efficiency, rapid response is crucial. Therefore, electrochemical methods are still the mainstream in disease screening scenarios where timeliness and clinical reliability are required.

## 4. Figure of Merits for the Performance Evaluation of Single-Ion Wearable Sensors

Wearable devices for single-ion monitoring in perspiration have undergone rapid evolution, with performance optimization remaining at the center of contemporary research. These platforms are primarily deployed for the longitudinal monitoring of sports physiology and the pre-screening of associated pathologies [92,93]. Depending on specific clinical or athletic scenarios, these sensors must fulfill strict performance indicators and analysis criteria. Nevertheless, a substantial technological gap persists between current ionic sensing capabilities and the idealized requirements of personalized wearable applications, necessitating sustained research investment. Table 2, Table 3, Table 4 and Table 5 consolidates the primary research milestones and advancements achieved in this domain over recent years.

### 4.1. Sensitivity

In electrochemical sensing, sensitivity is defined as a ratio of the response signal to the change in the concentration of the analyte. The specific quantification depends on the sensing mechanism. For potentiometric ISEs, sensitivity is expressed in the Nernstian slope (mV/dec), which is the slope of the linear relationship between electrode potential and the logarithm of ion activity under open-circuit conditions. When the reported sensitivity for a given ion exceeds the theoretical Nernst value [45,68], it generally indicates a deviation from thermodynamic equilibrium, possibly arising from coupled ion–electron transfer, membrane failure, or redox interference. It is worth noting that this phenomenon is not a sign of superior performance, but rather a sign of poor selectivity or unstable signal. For amperometric sensors, sensitivity is defined as the change in current per unit concentration (μA/mM), obtained from the slope of the steady current versus concentration calibration curve. For transistor-based sensors, sensitivity is the relative change in channel current with respect to the logarithm of analyte concentration.

To augment sensitivity, researchers have utilized novel materials with superior electrical conductivity and mechanical robustness to architect responsive sensing layers. For instance, shape-memory polymers and dynamically crosslinked hydrogels offer potential for self-healing and extended operational lifespans under environmental stress, thereby enabling more reliable and intuitive health monitoring [94,95]. Hybrid materials, such as MXene-polymer composites and metal–organic frameworks (MOFs), provide advanced electronic properties and biomarker-specific recognition to address the increasing demands for analytical precision and multifunctionality [96,97].

It is also noted that the r-SENSER sensor [98] exhibited a detection range of 10–160 mM for Na^+^. Notably, the lower bound of this range is only marginally above the physiologically relevant minimum (~10 mM), whereas under pathological conditions, sweat Na^+^ concentrations may fall as low as 5–10 mM. Consequently, the sensor is incapable of reliably measuring ion concentrations at or below borderline. This indicates the need to broaden the detection range in the future to ensure consistency with the physiological concentration of the analyte of interest, facilitating the clinical application of wearable sweat ion sensors.

### 4.2. Stability

Stability is defined as the capacity of a sensor to maintain consistent and reliable analytical performance over extended operational periods. It is typically characterized by the potential drift rate and inter-batch variability. The potential drift rate is commonly expressed in mV·h^−1^, with typical ISEs exhibiting drift values in the range of 0.1–1 mV·h^−1^. The formation of an interfacial water layer and suboptimal adhesion of the ISM to the electrode surface induce mechanical instability and subsequent potential drift [99]. Dai et al. [51] introduced a SnS_2_-MoS_2_ heterojunction as the ion-to-electron transduction layer and achieved an exceptionally low drift rate of 1.37 μV·h^−1^ over 24 h under rigorously controlled experimental conditions. However, this performance has not been validated in complex sweat matrices, raising concerns about its reproducibility.

Batch-to-batch reproducibility (CV%), in contrast, describes the degree of performance variation among sensors fabricated in different production batches, reflecting the controllability of the manufacturing process and the feasibility of large-scale production. However, CV% is rarely reported in most studies on wearable sweat sensors. This suggests that the majority of these devices remain at the laboratory stage and have not achieved the manufacturing maturity required for commercial deployment and clinical application.

Other than for high-sensitivity OFETs, the semi-crystallinity and molecular chain disorder of the semiconductor layer often result in suboptimal charge carrier mobility and unstable electronic characteristics. Compared with traditional inorganic semiconductors, the lack of standardized protocols for organic polymer synthesis renders these devices highly susceptible to experimental variability and structural defects. Consequently, future efforts must focus on fundamental materials research and the standardization of fabrication processes to enhance the yield and performance reproducibility of OFET platforms [100]. For colorimetric sensors, light source intensity attenuation can cause baseline drift. In addition, the colorimetric method based on the enzymatic reaction mechanism has poor batch-to-batch repeatability and is difficult to standardize on a large scale. In contrast, photonic crystal sensors with tunable optical properties for electrolyte detection have been reported, showing potential for the robust monitoring optically of ion changes [101].

### 4.3. Reliability

Optical colorimetric sensors have achieved significant milestones in substrate engineering (e.g., paper, textiles, hydrogels, and PDMS), microfluidic design (integrating pumps, Tesla valves, and check valves), and data acquisition modalities (leveraging smartphones and standardized color charts). While colorimetry offers an intuitive and convenient readout mode, its analytical accuracy and reliability remain challenged by multiple operational factors.

Chromogenic uniformity is a major concern, under conditions of insufficient sweat volume or constrained reaction kinetics, and inadequate mixing between reagents and samples can elicit non-homogeneous color distribution within the detection zone. Furthermore, in paper-based substrates, intrinsic capillary effects and the “coffee-ring” phenomenon induce uneven pigment distribution, leading to significant fluctuations in digital RGB extraction [102,103]. Reagent leaching and uncontrolled diffusion further cause inconsistent spatial color responses, thereby diminishing quantification accuracy. From a future perspective, advanced micro/nanofabrication techniques, including photolithography, laser engraving, 3D printing, and digital light processing (DLP), are required to engineer sophisticated microfluidic pumps and valves. Such enhancements facilitate regulated sweat transport, minimize evaporative loss, and preclude backflow contamination, thereby augmenting analytical precision [36,104]. Similarly, electrochemical sensors based on ISMs face the challenge of interference effects, where cross-selectivity from coexisting ions in sweat may cause systematic overestimation or underestimation of measurements. To address this issue, future researchers may explore algorithmic approaches to enhance data reliability.

Colorimetric readouts are inherently vulnerable to ambient light interference. Quantification typically involves extracting RGB coordinates from digital images and establishing linear or non-linear correlations (via chromaticity diagram calibration) with target concentrations. However, fluctuations in ambient light intensity and color temperature significantly distort the fidelity of extracted chromatic features. Additionally, colorimetric data acquisition is inherently discrete [105]. Unlike the continuous monitoring capability of electrochemical platforms, colorimetric analysis is limited by the sampling frequency. While algorithmic corrections and structural optimizations are being actively explored to address these issues [106], high precision detection within complex lighting environments and low-volume sample regimes remains a critical area for future breakthroughs.

### 4.4. Response Time

Response time is defined as the duration required for a sensor, upon exposure to an analyte, to reach a steady-state output signal, typically 90% or 95% of the final value. The typical response time of electrochemical sensors is 10–60 s. ISEs typically operate on a second-scale response, which is broadly consistent with iontophoresis-induced sweat secretion, thereby satisfying the requirements of routine sweat analysis. However, in scenarios involving rapid dynamic changes (e.g., rapid fluctuations in ion concentrations at the onset of exercise), the second-scale response may be insufficient to resolve transient peaks. In contrast, optical sensors based on nanozymes or immobilized chromogenic reagents typically require 2–20 min to reach a stable signal. In the sweat test induced by pilocarpine ion electroosmosis, the sweat composition changes rapidly in the initial stage, and minute-level sensors are insufficient to capture dynamic changes. Consequently, colorimetric sensors are more suitable for end-point measurements (e.g., post-exercise sweat patch analysis), which limits their applicability in continuous monitoring and clinical settings.

Transistor-based sensors can achieve response times as fast as the millisecond scale. However, in OECTs, device performance involves a dilemma between response time and sensitivity, and accelerating the response typically requires reducing the channel thickness, which diminishes ionic doping and lowers sensitivity. Although OECTs achieve millisecond-level responses and hold promise for real-time monitoring, their practical advantage is constrained by the intrinsic timescale of sweat secretion. As a result, their ultrafast response cannot be fully leveraged in user-oriented daily monitoring scenarios.

## 5. Cases of Wearable Multi-Ion Sweat Sensors

The limitations of single-ion sensors in reliably interpreting complex physiological states arise from the multifactorial regulation of sweat ion concentrations by factors such as perspiration rate, thermoregulation, neuromuscular exertion, and endocrine stress. These sensors are vulnerable to confounding influences including sweat rate fluctuations, evaporative loss, localized contamination, and mechanical deformation-induced signal drift during intense physical activity, which collectively compromise analytical fidelity. Multi-ion sweat sensing platforms that simultaneously quantify three or more ionic species on a single device have emerged to address these challenges by leveraging cross-calibration mechanisms that enhance reliability and discriminatory power compared to single-analyte devices. Advances in multiplexed architectures focus on streamlined circuit designs, improved system integration, and enhanced algorithmic signal processing to achieve robust real-time monitoring of exercise-induced electrolyte loss [107,108,109,110]. Such wearable platforms include flexible organic transistor arrays with ion-selective membranes, fabric-based multi-channel sensors activated in magnetic fields [111], and fully integrated wristwatch systems employing hierarchical porous carbon electrodes for stable multi-ion detection [112,113]. These developments demonstrate superior sensitivity, selectivity, and stability for simultaneous monitoring of key ions like sodium, potassium, and calcium in sweat, enabling more accurate assessment of physiological status during dynamic conditions [114,115,116,117]. Current research trajectories have increasingly pivoted toward achieving robust multi-ion sensing by streamlining circuit architectures, optimizing system integration strategies, and augmenting the precision of algorithmic classification and signal processing.

### 5.1. Sensing Mechanism of Multi-Ion Sweat Sensor

Advances in microfabrication technology have enabled multiple integration strategies for multi-ion detection devices. Among these, electrochemical arrays allow for simultaneous multi-ion detection through shared reference electrodes. In addition, in scenarios where rapid response is not critical, colorimetry offers a simple and effective alternative. And the organic transistor arrays further promote device miniaturization. Moreover, the combination of electrochemical and colorimetric methods enables both real-time electrical signal output and direct visual readout, combing quantitative accuracy and operational flexibility.

**Figure 3:**
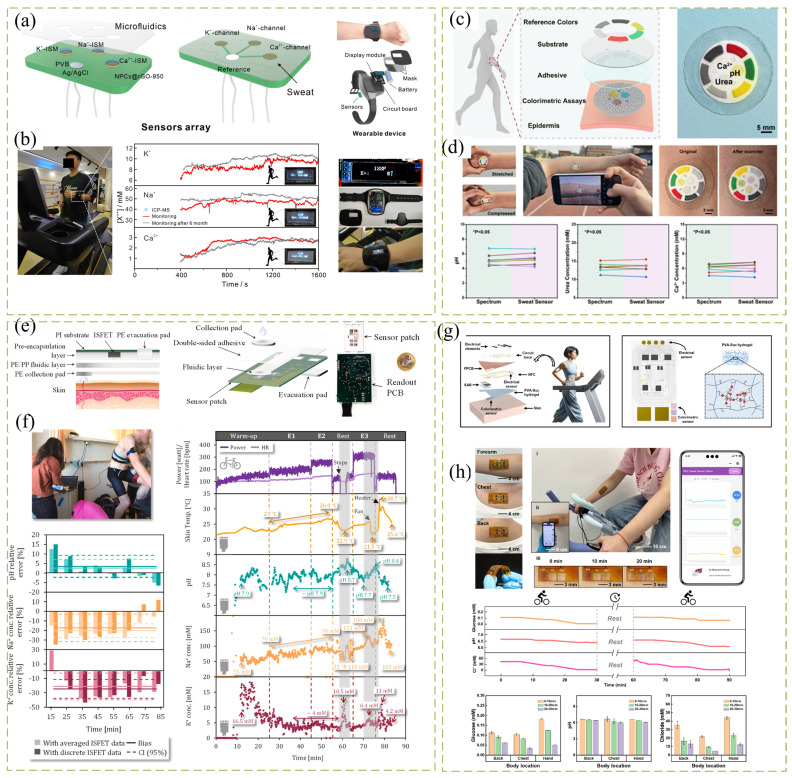
The detection principle and on-body verification of multi-ion sweat sensors. (**a**,**b**) Electrochemical detection of Na^+^, K^+^, and Ca^2+^ [110]. Copyright 2024. Reproduced with permission from American Chemical Society. (**c**,**d**) Colorimetric detection of Ca^2+^, pH, and urea [116]. Copyright 2024. Reproduced with permission from American Chemical Society. (**e**,**f**) Transistor-based detection of Na^+^, K^+^, and pH [117]. Copyright 2024. Reproduced with permission from Elsevier. (**g**,**h**) Integrated colorimetric and electrochemical detection of Cl^−^, pH, and glucose [85]. Copyright 2024. Reproduced with permission from Wiley-VCH.

Considerable progress has already been made in sweat sensors based on electrochemical methods to realize real-time monitoring of biomarkers in terms of designing stable solid contact interfaces and fully integrating multiple modules for large-scale applications of sweat sensors [110]. As illustrated in Figure 3a, such systems can share a single reference electrode, thereby enabling device miniaturization. On the other hand, colorimetric sensors enable cost-effective fluid transport through designed microchannels, allowing the construction of multiple parameter sensors on flexible interfaces (Figure 3c).

Furthermore, with advances in microfabrication and nanofabrication technologies, transistor-based sensors offer new technical pathways for multi-ion detection. (Figure 3e) Nevertheless, for ISFETs, the accumulation of sweat at the skin–device interface remains a major challenge to their continuous monitoring capability [116]. Moreover, critical issues such as power supply and skin-interface compatibility continue to limit the application of wearable sweat monitoring devices in personalized healthcare and early disease detection. He et al. [85] proposed the integration of colorimetric and electrochemical sensor arrays, as shown in Figure 3g,h. This multimodal sensing approach provides sufficient capability to support real-time monitoring of metabolic biomarkers in sweat, with the ability to visually observe changes in the colorimetric sensors for long-term data monitoring. Over time, the sensitivity of electrochemical sensors may degrade, potentially compromising the accuracy and reliability of biomarker detection. Additionally, the hydrogel components essential for the contrasting color platforms may encounter stability issues under varying environmental conditions, which could affect the system’s responsiveness. Future efforts should focus on developing materials with rapid response, good biocompatibility, and low cost while maintaining flexibility, in order to advance the development of multi-ion detection platforms.

### 5.2. Modularly Reconfigurable Architectures for Personalized Health Monitoring Platforms

**Figure 4:**
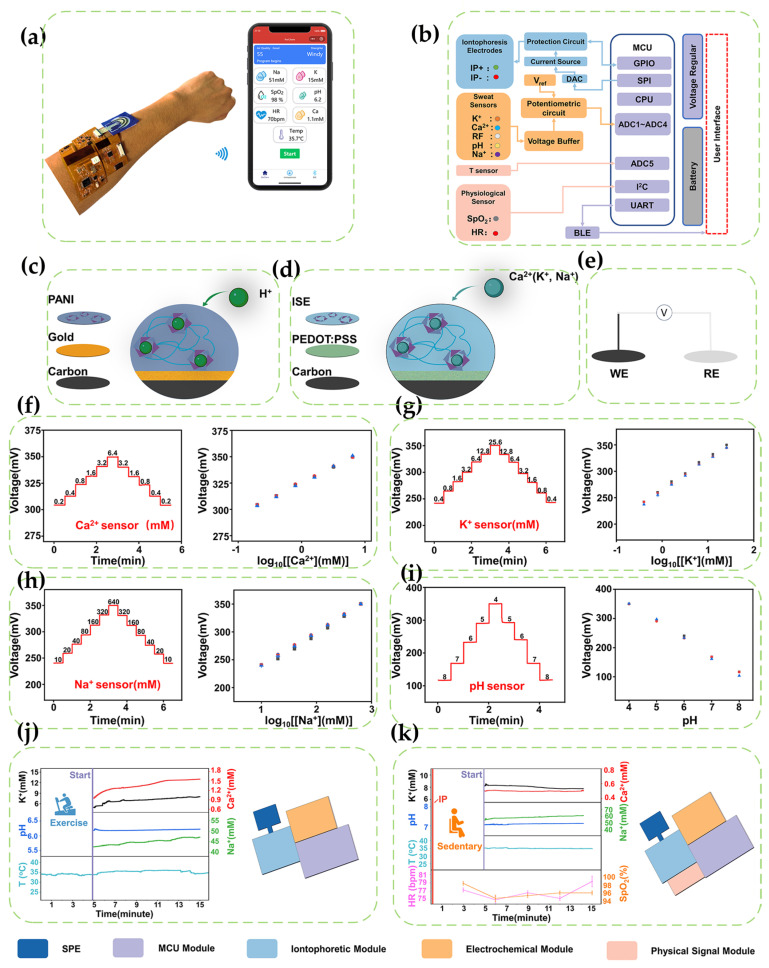
Monitoring of sweat ions via a reconfigurable modular system. (**a**) Picture of ReModuWear on the skin and a demonstration of the mobile phone application for real-time tracking. (**b**) Block diagram illustrating the relationship between the modular circuit and its associated connections, with each color representing a different module. (**c**,**d**) Schematic diagram of the exploded view of the working electrode of the (**c**) pH sensor and (**d**) Ca^2+^ (K^+^, Na^+^) sensor. (**e**) Demonstration of the potentiometric method for sensors. (**f**–**i**) Potentiometric response and linear fit (*n* ≥ 3) of four types of ionic sensors, namely, (**f**) Ca^2+^, (**g**) K^+^, and (**h**) Na^+^ sensors in deionized water and (**i**) the pH sensor in McIlvaine buffer. (**j**,**k**) On-body evaluation of the two types of wearable devices via various structural approaches. Results of test data using a configurable manner of sweat detection in exercise (**j**) and sedentary (**k**) state [118]. Copyright 2024. Reproduced with permission from the American Chemical Society.

Given the intricate correlations between biophysical signals, including body temperature and heart rate, and biochemical markers, alongside the significant thermal influence on electrochemical transduction, there is an urgent demand for the development of multimodal sensing platforms capable of concurrent physicochemical monitoring. Lin et al. [118] proposed a strategy based on modular circuit assembly for the customization of health monitoring wearable devices. The system targets four key sweat biomarkers (K^+^, Ca^2+^, Na^+^, and pH) and three essential physiological indicators (heart rate, blood oxygen, and skin temperature) as detection objectives (Figure 4a).

The transition from single-ion to multi-ion monitoring often requires intricate customization by designers and imposes additional costs on users. The system consists of four independent modules, which can be assembled on demand (Figure 4b): a microcontroller (MCU), an electrochemical detection module, an iontophoresis stimulation module, and a physical signal module. The electrochemical module utilizes screen-printed electrodes (SPEs) modified with specific ion-selective membranes (ISMs) and a transduction layer (PEDOT:PSS/Gold/PANI) (Figure 4c,d). Experimental data show good linear relationships for Ca^2+^ (0.2–6.4 mM), K^+^ (0.4–25.6 mM), Na^+^ (10–640 mM), and pH, with slopes of 30.44 ± 0.37 mV/decade, 59.32 ± 0.71 mV/decade, 60.66 ± 0.16 mV/decade, and 59.75 ± 1.07 mV/pH, respectively (Figure 4f–i).

In subsequent application scenarios, ion sensors exhibited signal fluctuations at the initial stage of detection during cycling (Figure 4j), which gradually stabilized as the detection progressed. The data further indicate that as sweat secretion increases, sweat pH tends to shift toward alkalinity. In contrast, during resting states (Figure 4k), all four ion sensors demonstrated consistent electrical signal stability. This reconfigurable modular wearable system (ReModuWear) is expected to provide users with a user-friendly interface and comprehensive health assessment solution. However, the proliferation of discrete sensing units often necessitates an expanded footprint of rigid circuitry, which fundamentally compromises the device’s mechanical stretchability and conformal skin interfacing. Such mechanical mismatches can elicit lateral sweat leakage or uncontrolled evaporation, thereby introducing substantial analytical errors in analyte concentration detection. In addition, consistent with single-ion potentiometric platforms, the precision of these integrated devices remains fundamentally constrained by the theoretical Nernstian sensitivity limit. Moreover, achieving sustainable power delivery for continuous, longitudinal monitoring without augmenting the overall system volume represents a persistent bottleneck that must be addressed.

### 5.3. High-Density Graphene Arrays Integrated Sensing Platform Enhanced by Machine Learning Classification

**Figure 5:**
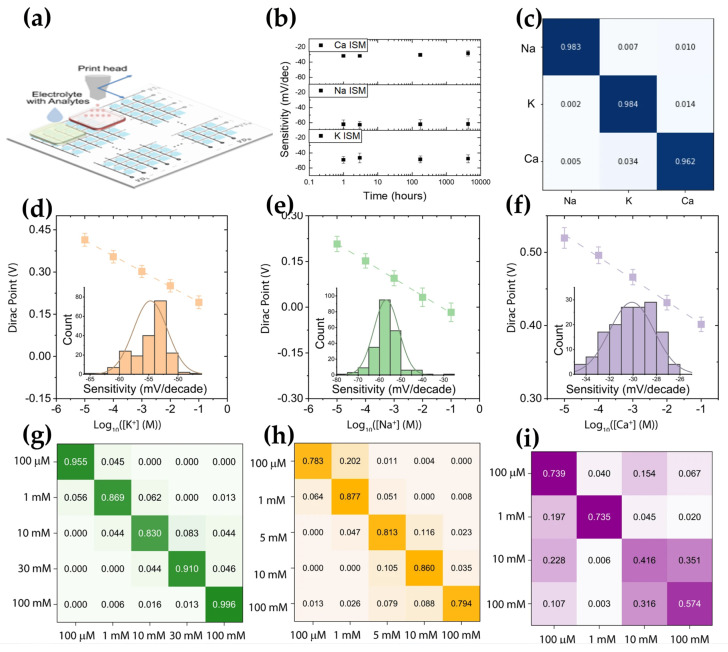
Multiplexed ion-sensing platform based on high-density graphene field effect transistor (GFET) arrays and machine learning enhancement. (**a**) Schematic of the sensing chip consisting of 16 × 16 sensor units. (**b**) Long-term stability assessment of the functionalized sensors over a 6-month period. (**c**) Confusion matrix for ion-type classification based on array-level response features. (**d**–**f**) Representative concentration-dependent shifts in transfer characteristics for K^+^ (**d**), Na^+^ (**e**), and Ca^2+^ (**f**) sensing, respectively. (**g**–**i**) Confusion matrices for concentration prediction of K^+^ (**g**), Na^+^ (**h**), and Ca^2+^ (**i**) across multiple concentration ranges using array-derived features and data-driven analysis [119]. Copyright 2022. Reproduced with permission from Springer Nature.

Current wearable multiplexed sensing platforms are frequently hindered by compromised analytical reliability, stemming from intrinsic device heterogeneity and signal crosstalk. To address these limitations, Xue et al. [119] developed an integrated sensing platform utilizing high-density graphene field effect transistor (GFET) arrays for the in situ monitoring of Na^+^, K^+^, and Ca^2+^ in perspiration. Diverging from conventional methodologies focused on discrete device optimization, this architecture integrates a 16 × 16 array on a monolithic chip, leveraging sensor redundancy to effectively counteract the fabrication variations inherent to two-dimensional materials (Figure 5).

The platform incorporates three distinct ISMs for Na^+^, K^+^, and Ca^2+^, integrated via high-precision material jet printing. Following array averaging processing, the system sensitivities for all target species converged toward their theoretical Nernstian limits (Na^+^: 56.8 mV/dec; K^+^: 54.7 mV/dec; Ca^2+^: 30.1 mV/dec, Figure 5d,e). Furthermore, coupled with a 6-month longitudinal stability assessment (Figure 5b), this underscores the exceptional interfacial robustness of the array within complex electrolytic environments. To streamline calibration, the author also pioneered a profile matching strategy, utilizing the statistical signatures of array-derived current slices instead of comprehensive curve scans. When processed via Random Forest algorithms, this approach achieved a 97.6% accuracy in ion-type classification (Figure 5c). In mixed analyte evaluations, the model delivered high concentration prediction accuracies of 90.6% and 82.6% for K^+^ and Na^+^, respectively (Figure 5g–i), and with a relatively diminished performance for Ca^2+^ (61.7%). Providing a robust algorithmic framework for mitigating complex background interference in sweat, the prohibitive fabrication costs precluded the validation of this platform in practical wearable on-body trials. Moving forward, the development of scalable, cost effective architectures remains a critical prerequisite for the widespread realization of multiplexed perspiration ionic analysis.

## 6. Challenges and Strategy

The above studies demonstrate that multi-ion, multiple-parameter wearable sweat sensors hold substantial potential for real-world applications. These advances are driven by improvements in multiplexed sensing platforms, enhanced biofluid sampling strategies, and progress in flexible materials and wireless electronics [120,121,122]. These developments have improved the reliability, analyte monitoring capability, and wearability of biosensors. However, the state of the art in this field remains at the stage of demonstrating proof-of-concept wearable platforms for detecting several representative biomarkers, and still faces many fundamental challenges in practical applications.

### 6.1. Lack of Adequate Physiological Evidence

Establishing the concentration of each ion and its relationship with blood chemistry and specific medical conditions is critical for expanding the impact of wearable sweat monitoring technologies in healthcare and for achieving their broad clinical acceptance [123]. For many analytes (e.g., Ca^2+^, NH_4_^+^), the correlation between their concentrations in sweat and in blood has not been fully clarified. A clear understanding of the physiological mechanisms underlying ion concentration changes, as well as real-time correlation between analyte levels in sweat and blood, is essential for clinical applications.

Future studies should focus on establishing a reliable sweat–blood transfer function, enabling accurate inference of physiological states from sweat-based measurements, and promote the clinical diagnostic value of sweat-derived biomarkers [124].

### 6.2. Clinical Validation and Regulatory Approval

Current studies have limited population sample sizes to validate application scenarios of sensors, far from meeting the standards required for clinical validation [125,126]. To enable successful application of wearable sweat sensors in the medical field, large-scale clinical studies are essential, along with aligning data analysis metrics with established clinical gold-standard diagnostic criteria for relevant diseases. Such large-cohort investigations and standardized evaluation frameworks are prerequisites for developing reliable and safe wearable ion-sensing diagnostic platforms. Furthermore, the use of wearable devices for disease screening, diagnosis, or monitoring must comply with medical device regulatory requirements (e.g., FDA/CE), reducing device risk to an acceptable level.

### 6.3. Data Analysis and Privacy Protection

In recent years, artificial intelligence (AI) technologies have made significant advances in data analysis and mining [127], and have been increasingly applied in medical and physiological sciences. This is particularly evident in the analysis of the complex composition of sweat, where AI demonstrates substantial utility. The primary objective is to enable devices to perform continuous, real-time measurements, compute and predict trends, and thereby improve health conditions or optimize training patterns [128]. By employing AI-driven correction and learning algorithms, sweat biomarkers can be correlated with other key interacting factors (such as temperature and heart rate), generating data that are more closely associated with physiological states.

However, the opacity and lack of interpretability of algorithms in the calculation process constrain their direct use in clinical decision-making. In addition, if data storage and transmission do not comply with security standards such as HIPAA/GDPR, there is a significant risk of personal data breaches. Future efforts should focus on enhancing the real-time data support capabilities of device systems to meet both clinical and privacy protection requirements.

### 6.4. Translation to the Commercial Market

The global wearable sensors market size is projected to reach USD 9.26 billion by 2030, growing at a CAGR of 13.5% from 2024 to 2030 [129]. Wearable sweat ion sensors face numerous challenges related to their fundamental operation during the transition from experimental research to commercialization. To accommodate user requirements in complex scenarios (such as high temperature, high humidity, or intense physical activity), the hardware should not only achieve multiple parameter integration and lightweight design [130] but also ensure stable adhesion to the skin without causing additional irritation, enabling robust performance across diverse scenarios without frequent recalibration [84,131,132]. In addition to optimization of technical performance itself, high fabrication costs, limited large-scale manufacturing capability, and insufficient biosafety evaluation also constitute key barriers to their societal acceptance and market adoption.

Considering all the above challenges, promoting wearable sweat ion sensors from the laboratory to clinical and commercial applications requires coordinated efforts among researchers, end users, and market stakeholders, and a systematic approach to overcoming these practical issues.

## 7. Conclusions

This review systematically summarizes recent advances in wearable sweat ion sensors. These sensors have developed from single-ion detection systems into integrated multifunctional platforms capable of simultaneously quantifying multiple ions as well as biomarkers such as glucose, lactate, and cortisol, demonstrating significant multiplexing capabilities. Currently, the predominant detection mechanism is electrochemistry, which, combined with advanced nanomaterials [133], precision fabrication techniques [134], and artificial intelligence [2], has effectively enhanced sensor sensitivity, stability, reliability, and response speed. Regarding multi-channel construction, electrochemical arrays, microfluidic colorimetric patches, and their combination represent the main integration strategies. Furthermore, through hardware integration and algorithmic optimization, the accuracy of sensors for detection in complex environments has been further enhanced. However, for practical applications in clinical diagnosis and long-term daily health monitoring, wearable sweat ion sensors still address several key challenges, including validation of the correlation between sweat and blood ion concentrations, large-scale clinical sample validation, data security and privacy risk control, and the development of scalable manufacturing processes. Addressing these issues is a necessary prerequisite for meeting regulatory requirements and advancing the development of this field.

## Figures and Tables

**Table 3:** Monitoring of Ca^2+^ in sweat.

Ions	Methods	Substrate	Electrode Fabrication	Electrode Material	Transducer	ISM Polymer	Reference Membrane	Detection Range (mM)	Sensitivity	Response Time	Literature
Ca^2+^	Extended gate OFET (ExG-OFET)	PET	Photolithography, Chemical etching, Drop-casting	Au (gate)	Organic semiconductor (DPP-TTT)	PVC	Ag/AgCl	1.6 μM (LOD)–18 mM	>60 mV/decade	3 ± 1 s	[45]
Ca^2+^, Na^+^	Potentiometric	Polyurethane/silk fibroin complex film	Magnetron sputtering	Cr/Au	PtPRGO (Polydopamine functionalized RGO with Pt NPs)	PVC	Ag/AgCl	Ca^2+^: 0.25–2; Na^+^: 1–1000	Ca^2+^: 91.98 mV/dec Na^+^: 88.58 mV/dec	Real-time	[75]
Ca^2+^	Potentiometric	PET	Screen printing	Carbon ink	Carbon ink	PVC	PVB/NaCl	0.5–4	30.6 mV/decade	<5 s	[76]
Ca^2+^,	Potentiometric	Cotton fabric	Screen printing, Laser cutting (ABS film for microfluidics)	Carbon ink	Carbon ink	PVC	PVB/NaCl	0.125–2	30.46 mV/decade	<5 s	[77]
Ca^2+^	Potentiometric	PET	Dispense printing, 3D printing (molds for microfluidics)	Au	PEDOT:PSS on Au dendrites	PVC	PVB	0.1–100	31.41 mV/decade	Real-time	[78]

Note: The LOD was determined as the calcium ion concentration corresponding to the intersection of the linear regression line with the voltage equivalent of three times the noise level. For the purpose of comparison in this review, “Real-time” is quantized as a response time < 30 s in this table.

**Table 5:** Monitoring of Cl^−^ and pH in sweat.

Ions	Mechanism	Substrate	Data Analysis	Indicator Dye	Detection Range (mM)	Response Time	Refs.
Cl^−^, pH	Colorimetric	PVA-Sucrose hydrogel	Smart phone	Cl^−^: silver chloranilate pH: chlorophenol red	Cl^−^: 10–100 pH: 3–9	N/A	[85]
Cl^−^, Ca^2+^, pH	Colorimetric	Ecoflex, cellulose membranes	Smart phone	Commercial	Cl^−^: 0–35 Ca^2+^: 0–15 pH: 3–8	N/A	[86]
Cl^−^, Fe^2+^	Colorimetric	Paper	Smart phone	Cl^−^: mercuric thiocyanate Fe^2+^: ferrozine solution pH: anthocyanin	Cl^−^: 20–100 Fe^2+^: 10–50 μM	N/A	[87]
Cl^−^, pH	Colorimetric	Fabric	Color sensor	Cl^−^: tripyridyltriazine (TPTZ) pH: mixed indicator (bromothymol blue, bromocresol purple, and thymol blue)	Cl^−^: 1–160 pH: 4–8	~30 s	[88]
Cl^−^, pH	Colorimetric	Cellulose film and a substrate of TPC	Smart phone	Cl^−^: silver chloranilate pH: anthocyanin	Cl^−^: 25–100 pH: 4.5–7	~15 min	[89]
Cl^−^	ECL	rGO@hydrogel Lum@hydrogel	Smart phone	N/A	1–800	N/A	[90]
Cl^−^, pH	Colorimetric	PDMS	Smart phone	Cl^−^: TPTZ pH: precise pH test strips	Cl^−^: 0.01 and 0.05 g·L^−1^ pH: 3.8–5.4	N/A	[91]
Cl^−^, Mg^2+^, pH	Colorimetric	PDMS	Smart phone	Cl^−^: mercuric thiocyanate Mg^2+^: chromium black T pH: bromocresol green, bromocresol purple, and bromothymol blue	Cl^−^: 5–160 Mg^2+^: 0.5–10 pH: 4.0–7.5	1 min	[49]

Note: ECL: Electrochemiluminescence. N/A, not applicable.

## Data Availability

The original contributions presented in this study are included in the article. Further inquiries can be directed to the corresponding author.
